# Self-administration of intravenous C1 esterase inhibitor (Berinertâ) in patients with Hereditary Angioedema decreases number of days spent in an emergency room

**DOI:** 10.1186/1710-1492-7-S2-A40

**Published:** 2011-11-14

**Authors:** Caroline Rizk, Stephanie Santucci, Sheryl McDiarmid, Jacob Karsh, William H Yang

**Affiliations:** 1 University of Ottawa, Ottawa, ON, Canada; 2 Allergy and Asthma Research Centre, Ottawa, ON, Canada

## Background

Hereditary Angioedema (HAE) is a rare, inherited, autosomal dominant disease caused by a deficiency in C1-esterase inhibitor. It affects one in every 50,000 to 100,000 individuals. There were no approved treatments for HAE in North America until 2009, when C1-esterase inhibitor (Berinertâ) was released. It is an intravenous medication that requires patients to present to an emergency room (ER) for treatment. We present two cases of patients with HAE who self-administered the medications, decreasing their number of emergency room visits substantially.

## Methods

Cases were obtained from office visits with an allergist and continued communication with the patients.

## Results

The first patient averaged around 17 visits to an ER per year. She and her husband are both paramedics and they were able to start intravenous lines for the required infusion at home. After she began self-administering the medication, she had no further visits to an emergency room. The second patient suffered from very frequent attacks and had poor venous access. In 2009, she had 37 ER visits and in 2010 alone, 48 visits. A Hickman catheter was placed in her internal jugular vein for easy access. After she began self-administration 5 months ago, she has had only 3 presentations to the ER.

## Conclusions

Self-administration of C1-esterase inhibitor (Berinertâ) dramatically improved the lives of these two young patients, and resulted in a considerable decrease in the number of days spent in an emergency room.

